# CRISPR/Cas9-mediated genome editing in naïve human embryonic stem cells

**DOI:** 10.1038/s41598-017-16932-y

**Published:** 2017-11-30

**Authors:** Eva Z. Jacobs, Sharat Warrier, Pieter-Jan Volders, Eva D’haene, Eva Van Lombergen, Lies Vantomme, Margot Van der Jeught, Björn Heindryckx, Björn Menten, Sarah Vergult

**Affiliations:** 1 Center for Medical Genetics, Ghent University, Ghent University Hospital, Ghent, Belgium; 2 Ghent Fertility and Stem cell Team (G-FaST), Department for Reproductive Medicine, Ghent University Hospital, Ghent, Belgium

## Abstract

The combination of genome-edited human embryonic stem cells (hESCs) and subsequent neural differentiation is a powerful tool to study neurodevelopmental disorders. Since the naïve state of pluripotency has favourable characteristics for efficient genome-editing, we optimized a workflow for the CRISPR/Cas9 system in these naïve stem cells. Editing efficiencies of respectively 1.3–8.4% and 3.8–19% were generated with the Cas9 nuclease and the D10A Cas9 nickase mutant. Next to this, wildtype and genome-edited naïve hESCs were successfully differentiated to neural progenitor cells. As a proof-of-principle of our workflow, two monoclonal genome-edited naïve hESCs colonies were obtained for *TUNA*, a long non-coding RNA involved in pluripotency and neural differentiation. In these genome-edited hESCs, an effect was seen on expression of *TUNA*, although not on neural differentiation potential. In conclusion, we optimized a genome-editing workflow in naïve hESCs that can be used to study candidate genes involved in neural differentiation and/or functioning.

## Introduction

Embryonic stem cells (ESCs) are derived from the inner cell mass (ICM) of developing pre-implantation mouse or human blastocysts and are characterized by their ability for self-renewal and pluripotency^1,2^. Since it’s shown that mouse pluripotent stem cells can also be derived from the mouse post-implantation blastocyst (epiblast stem cells, EpiSCs), two states of pluripotency are defined in mouse ESCs (mESCs) depicted as naïve and primed^3–5^. Naïve mESCs represent a more ground state of pluripotency in embryonic development and resemble cells of the pre-implantation epiblast *in vivo*. In contrast, EpiSCs resemble cells of the post-implantation epiblast, which is a later and already more advanced developmental stage, referred to as the primed state^3,5^. Naïve and primed mESCs show differences in several aspects such as colony morphology and active signalling-dependent pathways to maintain the pluripotent state, reflected in different transcriptomic and epigenetic characteristics^3,5,6^. In addition, naïve mESCs are able to survive single cell passaging, show a higher proliferation rate and have a higher clonogenic ability, compared to primed mESCs^5^.

Although human ESCs (hESCs) are also derived from the pre-implantation blastocyst, they differ from naïve mESCs in morphology, culture requirements, molecular profile, differentiation behaviour and clonogenicity^5^. Instead, these cells show more similarities to the post-implantation EpiSCs and thus conventional derived hESCs are considered as primed pluripotent stem cells^5,7^. In mouse, primed EpiSCs can be converted to the naïve state either by overexpression of pluripotency associated transcription factors or by applying naïve culture conditions. Hanna *et al*.^8^ were the first to convert primed hESCs into mESC-like hESCs by ectopic induction of key pluripotency factors. Later, other groups defined different naïve media conditions to induce naïve pluripotency in conventional hESCs^6,7,9–12^.

The ability of human ESCs to differentiate into cells of the three germ layers makes them an attractive model system to study human development and disease *in vitro* ^13–17^. To study gene function and the underlying regulatory pathways, genome editing has been shown to be of great avail^18–20^. The CRISPR/Cas9 (Clustered Regularly Interspaced Short Palindromic Repeats/CRISPR associated proteins) Type II system is originally a prokaryotic RNA-mediated adaptive immune system used as a defense against invading foreign genomes and adapted for easy and efficient genome-editing in virtually every desired genome^21,22^. Almost any DNA sequence of interest can be targeted with the help of a customizable single guide RNA (sgRNA) containing 20 base pairs complementary to the target site. This target site needs an adjacent protospacer adjacent motif (PAM) for recognition by the sgRNA. The Cas9 endonuclease is guided by the sgRNA to the target locus and will induce a double-stranded break 3 base pairs (bp) upstream of the PAM sequence. Either these breaks will be repaired by non-homologous end joining (NHEJ) often leading to the formation of insertions/deletions (indels) near the breakpoints as this process is error-prone, or an exogenous DNA sequence can be inserted during homology-directed repair (HDR)^21–23^. To avoid undesired off-target effects, caused by sequence homology between the sgRNA and off-target sites, a D10A Cas9 nickase mutant with paired guide RNAs can be used to enhance specificity^24^.

Compared to mESCs, conventional primed hESCs are more difficult to genetically manipulate and render some practical obstacles for efficient genome-editing^25^. A bottleneck with the culture of conventional hESCs is their low proliferation rate^7,26^, their inability for single-cell passaging by trypsin digest^7,25^ and the low single-cell cloning efficiency^7^. These characteristics complicate fast expansion of the culture and clonal isolation and selection after transfection^25^. This low single-cell cloning efficiency will also have an influence on the targeting efficiencies^27^. Another disadvantage of primed hESCs is the heterogeneity in differentiation potential^6,26^.

On the contrary, the naïve state enables fast proliferation and single-cell passaging. These are favourable characteristics to increase target efficiency, indicating this state is preferable for efficient genome-editing. It has indeed been reported that the naïve state is more amendable to genome-editing^7,25–27^. The study from Gafni *et al*.^7^ also suggests that HDR will be more efficient in naïve ESCs due to their more open chromatin state.

In the present study, we show an optimized workflow for genome-editing of naïve hESC using the CRISPR/Cas9 system followed by neural differentiation. As a proof-of-principle we generated a knockdown with CRISPR/Cas9 of the long non-coding RNA (lncRNA) *TUNA* that has already been described to be involved in neural differentiation^28^. Since induced pluripotent stem cells (iPSCs) of patients with a (chromosomal) rearrangement involving a particular gene, are not always available or cannot be generated, this workflow can be used to study candidate genes involved in neural differentiation and/or functioning.

## Results

### Efficient genome-editing in naïve hESCs with CRISPR/Cas9 system

**Figure 1:**
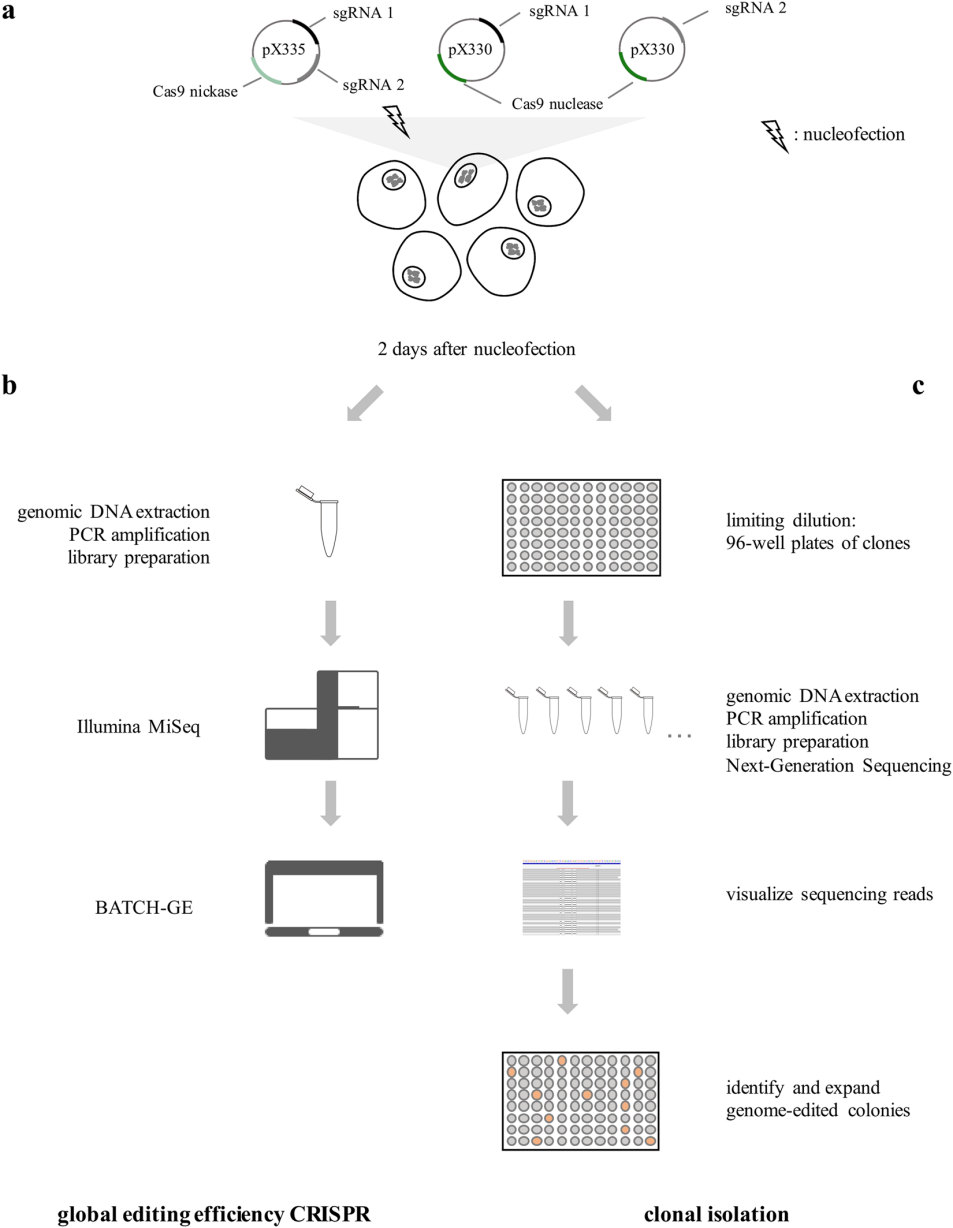
Optimized workflow for efficient genome-editing of naïve human embryonic stem cells. (**a**) Two sgRNAs were designed for each gene to allow double nicking and cloned in the pX335 plasmid. Both sgRNAs were also individually paired with the Cas9 nuclease (pX330 plasmid). The CRISPR plasmids were transfected in the naïve hESCs using nucleofection at day two after splitting. (**b**) To determine the overall editing efficiency of the CRISPR, DNA was extracted from the pool of transfected cells two days after nucleofection. After subsequent PCR and massive parallel sequencing (Illumina MiSeq), the overall editing efficiency and the detected indel variants were quantified with BATCH-GE^51^. (**c**) To obtain monoclonal genome-edited colonies, clonal isolation was performed two days after nucleofection, using limiting dilution (0.5 cells/100 µl). After 6–8 days, surviving clones were observed and DNA was extracted. After amplification and massive parallel sequencing of the target region, the sequencing reads were visualized in the Integrative Genomics Viewer (IGV, Broad Institute) to identify the genome-edited colonies.

To investigate the editing efficiency and characteristics of the CRISPR/Cas9 system in naïve hESCs, we nucleofected Cas9 nuclease as well as Cas9 nickase expressing plasmids targeting the coding gene *EMX1* and non-coding genes *TUNA* and *MEG3* in hESCs. The workflow is presented in Fig. 1. Each target site was successfully edited by both Cas9 proteins, however, we see big differences in editing efficiency, ranging from 1 to 19%.

**Figure 2:**
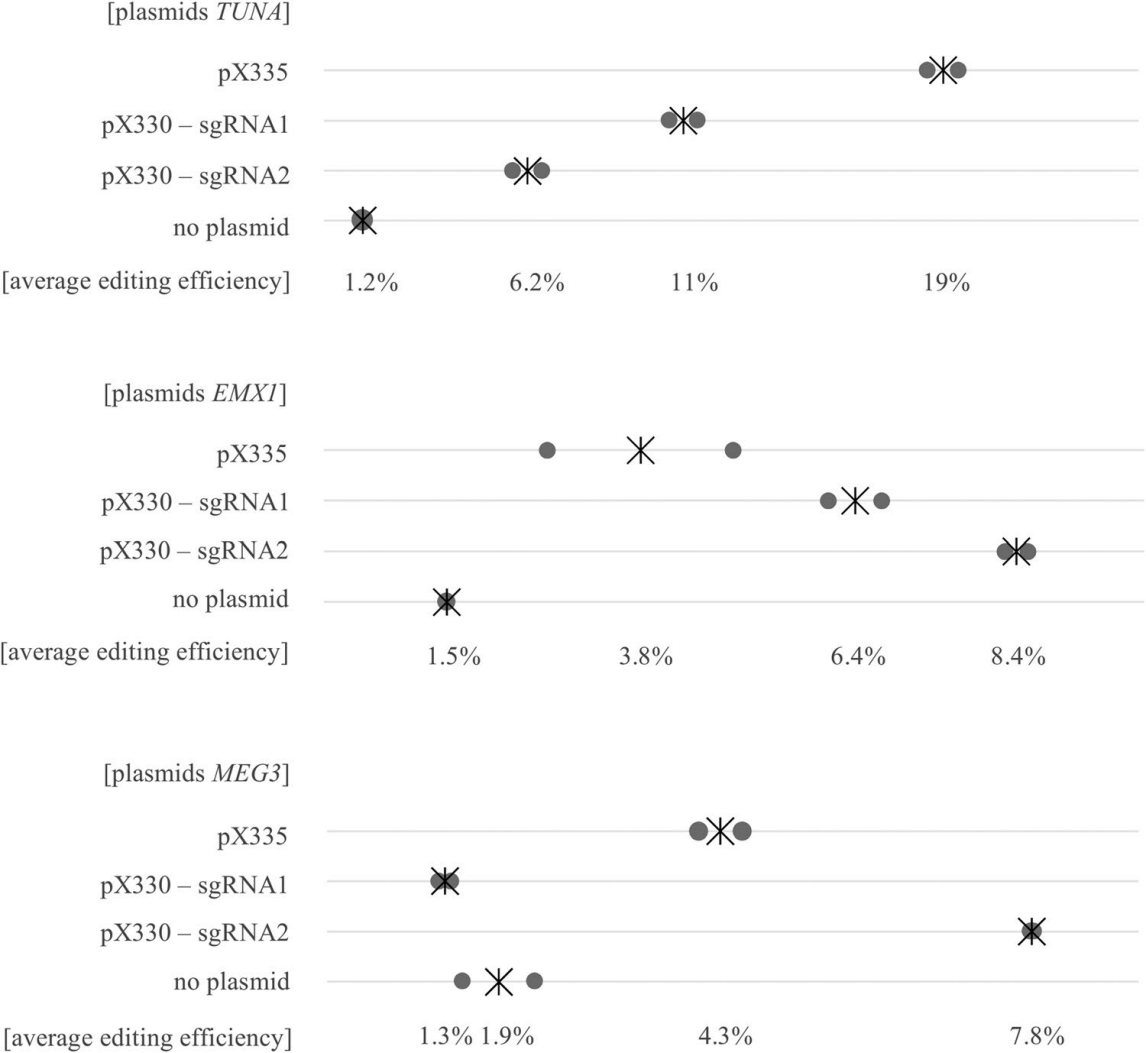
CRISPR/Cas9 works efficiently in naïve human embryonic stem cells. Editing efficiencies for *TUNA*, *EMX1* and *MEG3* after transfecting pX335, pX330-sgRNA1 or pX330-sgRNA2 plasmids in the naïve hESCs. As a control, mock-treated naïve hESCs (no plasmid) were used. Grey dots represent two biological replicates, the asterisk indicates the average of both replicates.

For *TUNA*, the highest editing efficiency (19%) was accomplished with the Cas9 nickase, however, for *EMX1* and *MEG3* the highest editing efficiencies, respectively 8.4% and 7.8%, were generated using the nuclease combined with one sgRNA (Fig. 2). Based on these three loci and taken into account that there is a certain variation in sgRNA activity, both the wildtype Cas9 nuclease and mutant Cas9 nickase can edit the on-target site efficiently at similar editing rates.

**Figure 3:**
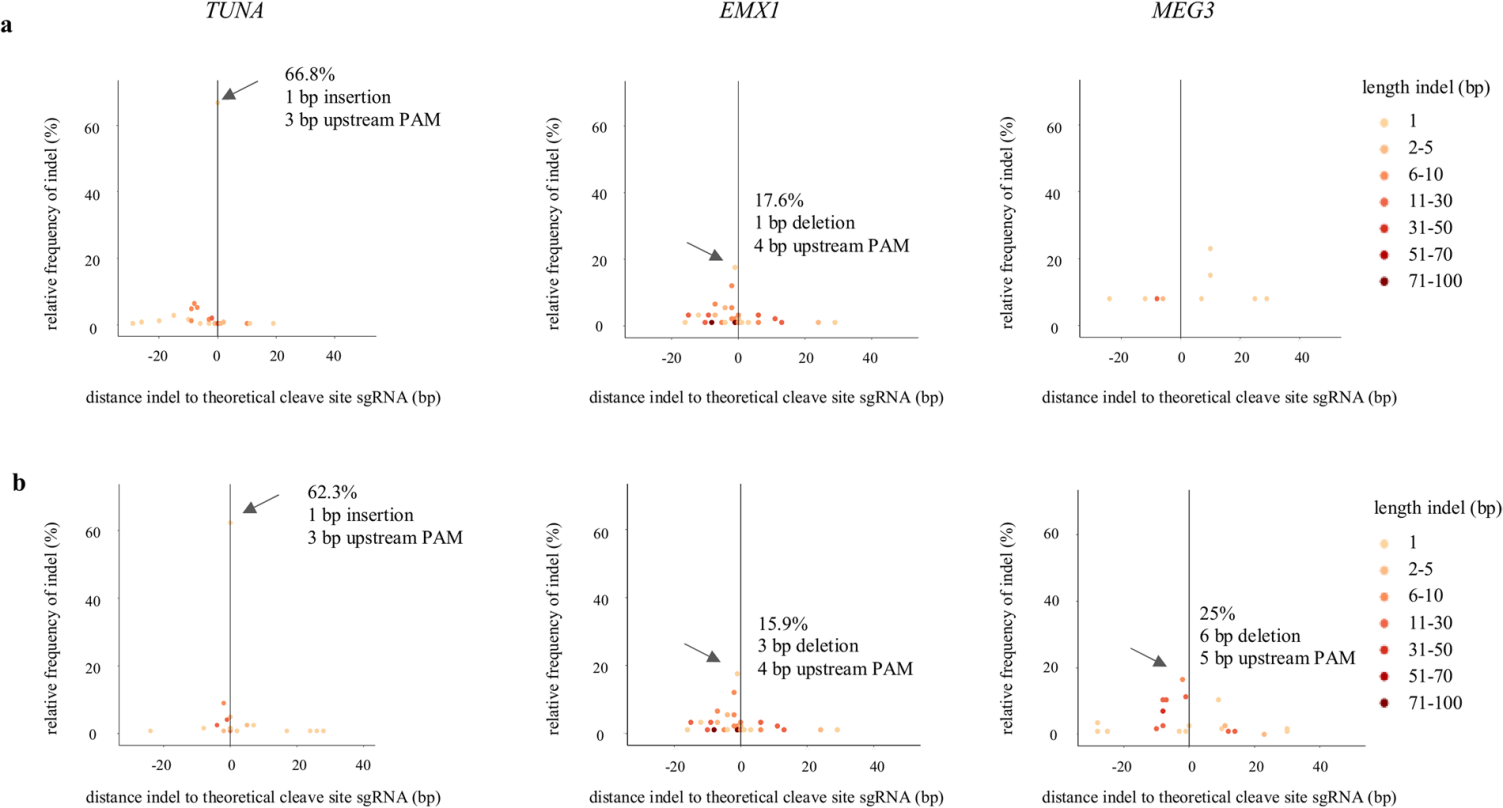
Cas9 nuclease generates predominant indels near the cleavage site. Distance of the start position of each insertion or deletion to the theoretical cleavage site (=3 bp upstream of the PAM sequence) is presented on the x-axis, while the relative frequency of the indel is presented on the y-axis for (**a**) nuclease – sgRNA1 and (**b**) nuclease – sgRNA2. Length of indels are represented by the different colors. Predominant indels are indicated with an arrow. Only 1 replicate is shown.

Mali *et al*.^29^ described in HEK-293T cells that most indels resulting from NHEJ after Cas9 nuclease activity are present near the 3′ end of the target site, particularly ∼3–4 bp upstream of the PAM, consistent with the theoretical cleavage site of Cas9. Also in the naïve hESCs we see that most indels are located around this position after editing with Cas9 nuclease. For *TUNA*, *EMX1* and *MEG3* predominant indels of 1–6 bp are present 3–5 bp upstream of the PAM sequence (Fig. 3). Remarkably, the same predominant indels are also present in HEK-293T cells with similar editing efficiencies as in the naïve hESCs (Supplemental Figs 1 and 2).

For *MEG3-*sgRNA1 however, only a few variants are located near this expected cleavage site (Fig. 3), suggestive of a none to very low active sgRNA in the naïve hESCs. Nonetheless in HEK-293T cells, the same sgRNA also shows low editing efficiency (2.2%), but a predominant indel is present at 3 bp upstream of the PAM sequence (Supplemental Figs 1 and 2).

**Figure 4:**
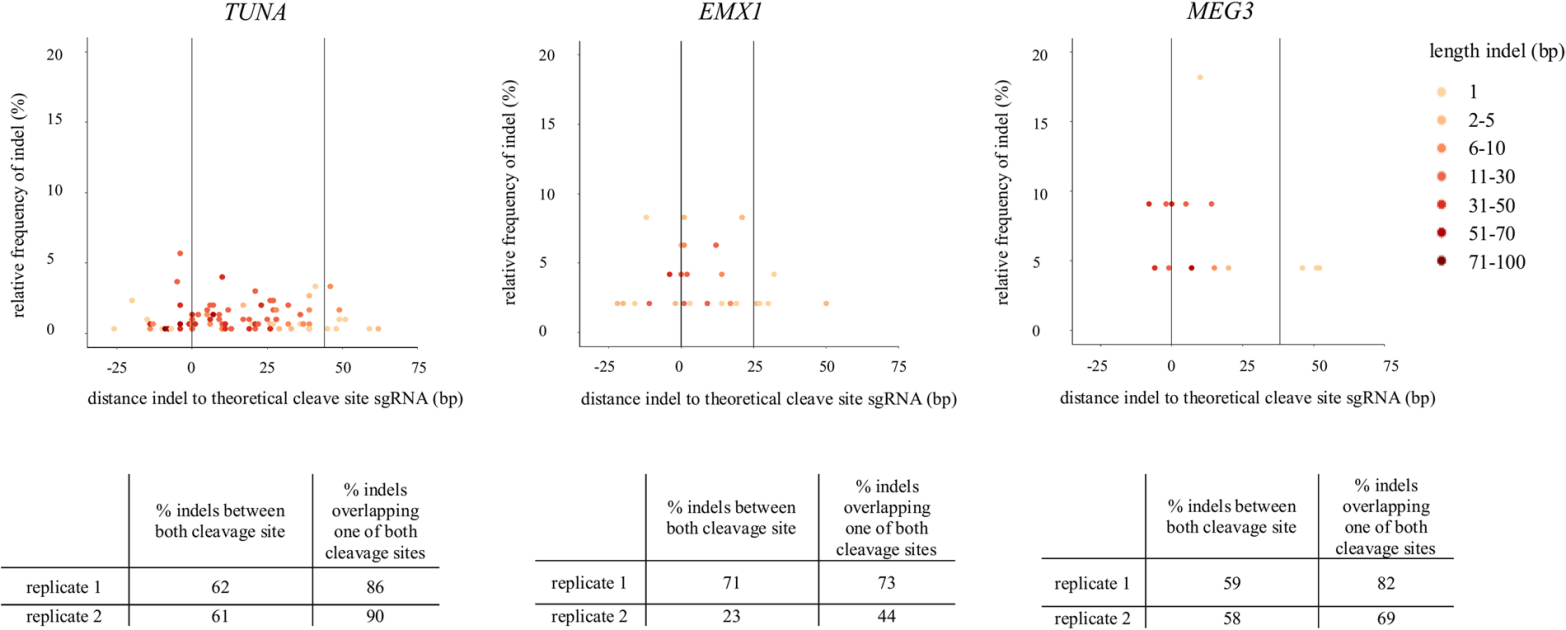
Majority of indels is present between both cleavage sites after editing with Cas9 nickase. Distance of the start position of each insertion or deletion to the theoretical cleavage site (=3 bp upstream of the PAM sequence) is presented on the x-axis, while the relative frequency of the indel is presented on the y-axis after editing with the Cas9 nickase. Length of indels are represented by the different colors. Only 1 replicate is shown. The majority of indels is present between the two theoretical cleavage sites (indicated by the black lines at position 0 (sgRNA1) and at positions 44 bp (*TUNA*), 25 bp (*EMX1*) and 38 bp (*MEG3*). Percentages of indels 1) within or spanning both the two cleavage sites and 2) indels spanning at least one of the two sites are shown below the graph. Replicate 2 of *EMX1* has an editing efficiency of 2.7% (compared to 5% for replicate 1), which can explain the lower percentage of indels located between the two cleavage sites.

After editing with the Cas9 nickase, no predominant indels are seen. Here, approximately 60% of the indels for *TUNA*, *EMX1* and *MEG3* are present completely between or spanning both the cleavage sites and 70–90% of indels span at least one of the two sites (Fig. 4). This is also the case in HEK-293T cells (Supplemental Fig. 3). These data demonstrate that our CRISPRs, both the Cas9 nuclease as nickase, cut at the target site.

After screening the top 4 highest ranked off-target sites for each sgRNA, no off-targets were detected for both sgRNAs for *TUNA* and *MEG3*. However, indels were present in one off-target region of *EMX1*-sgRNA2 in the cells cleaved with the Cas9 nuclease (12% editing efficiency), but not with the double nicking system (Supplemental Fig. 4). Since these undesired indels are completely intronic (first intron of *EXOC6)*, this off-target is unlikely to have a functional effect.

### Cas9 nickase generates larger indels compared to wildtype Cas9 nuclease

Based on median deletion and insertion sizes, indels generated with the Cas9 nickase are larger compared to the wildtype Cas9 nuclease (Supplemental Table 1). This is due to the creation of two nicks in each other’s neighbourhood compared to only one break point with the Cas9 nuclease. Since both sgRNAs for *EMX1* overlap six base pairs, making the two cleavage sites closer to each other compared to *TUNA* and *MEG3*, there is a smaller difference in deletion size between the Cas9 nuclease and nickase for this gene. For the nickase, a median size of 3 and 7 bp (rep 1 and 2) is seen and for both sgRNAs combined with Cas9 nuclease a median size of 6–8 and 3 bp (rep 1 and 2). The largest deletions were generated for *TUNA* (median size nickase 17 and 21 bp (rep 1 and rep2)) and the largest insertions for *MEG3* (median size nickase 5 and 25 bp (rep 1 and rep2)). Interestingly, NHEJ generates more deletions than insertions both after editing with the Cas9 nuclease as well as the nickase (Supplemental Table 1). Only for the Cas9 nuclease plasmids for *TUNA*, more insertions occur due to the predominant one base pair insertion at the cleavage site. The same indel characteristics are seen in HEK-293T cells (Supplemental Table 2).

### Successful generation of monoclonal genome edited naïve hESCs

Lin *et al*.^28^ reported *Tuna*, a lncRNA, to play an important role in pluripotency and neural differentiation of mESCs. As a proof-of-principle for our workflow, consisting of genome-editing in naïve hESCs followed by neural differentiation, we generated genome-edited hESC lines for *TUNA* using the double nicking system, as this system generated the highest editing efficiency combined with larger indels. The latter can be important as in contrast to coding genes, the functional effect of a certain indel is unpredictable for non-coding genes.

After transfection, the naïve hESCs were monoclonal isolated through limiting dilution in 96-well plates and a survival rate of 7% (7 out of 102 clones) was observed. The surviving clones were expanded and in the end, we obtained two monoclonal genome-edited *TUNA* colonies, one with a homozygous deletion and one with compound heterozygous deletions (Supplemental Fig. 5). To exclude that the latter is the result of a polyclonal colony, the clonal isolation was repeated and all resulting monoclonal colonies carry the same compound heterozygous deletions.

To confirm the naïve state of pluripotency after genome-editing, expression of transcription factors associated with either naïve or primed pluripotency was examined through qPCR in both genome-edited hESCs and compared to the wildtype naïve and primed hESCs (Supplemental Fig. 6a). We analyzed the expression of the naïve-specific pluripotency genes *KLF2* and *TCL1B* ^30^ in our primed and naïve stem cell lines and noticed a similar expression of both genes in the wildtype and genome-edited naïve hESCs and a trend of increased expression compared to primed hESCs. As expression analysis was performed on bulk cells, it is possible that certain naïve cells were already in a more differentiated state than others. This could explain the lower expression of *KLF2* and *TCL1B* in some biological replicates. No differences in expression of *NANOG* was seen between wildtype and genome-edited naïve hESCs, as well as between these naïve hESCs and their primed counterparts. This was however to be expected, as *NANOG* is a core-pluripotency factor with an important role in both the naïve and the primed state^31^. Next to this, in mESCs a variable expression of *Nanog* can be seen due to some heterogeneity in the mESC population^32^. This supports the assumption that at the time of RNA collection, a subpopulation of already differentiated cells was present in the naïve hESCs. De novo methyltransferase *DNMT3B*, a marker for the primed state, was clearly higher expressed in primed cells compared to all naïve cells^30^. Therefore, we can conclude that no differences in expression of naïve markers between the wildtype and genome-edited naïve hESCs were observed in our culture conditions. Moreover, there was no difference in morphology observed between the wildtype and genome-edited naïve hESC colonies (Supplemental Fig. 6b). These results indicate that the hESCs were still naïve after genome-editing.

### The generated deletions have an effect on both the secondary structure as well as the expression of *TUNA* in naïve hESCs

**Figure 5:**
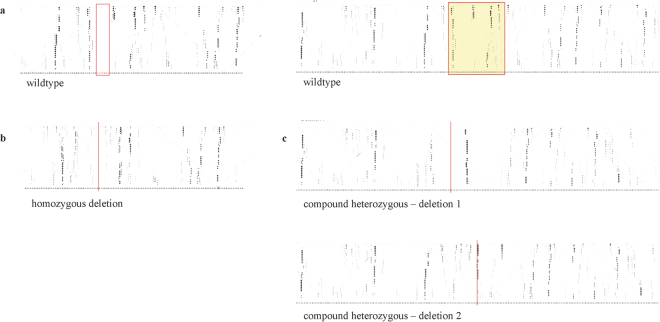
Local structures in the targeted region of *TUNA* are altered after genome-editing. Using RNAplfold, local averaged pair probabilities are computed and represented in a dot plot^33^. (**a**) Wildtype local RNA structure of *TUNA* (left red box: position of homozygous deletion; right red box: position of the compound heterozygous deletions). (**b**) and (**c**) respectively show the local RNA structure of *TUNA* in the colony with the homozygous deletion and in the colony with the compound heterozygous deletions after CRISPR/Cas9 editing. The red lines indicate the position of the deletions.

In comparison with the consensus RNA structure, both the homozygous as well as the compound heterozygous deletions showed a modification of the secondary structure of *TUNA*, which was assessed in silico using RNAplfold^33^. Fig. 5a–c respectively show the pair probabilities for the secondary structures of the wildtype RNA structure, the structure of the RNA with the homozygous deletion and the structure of the RNA with both deletions in the compound heterozygous edited cells. All three deletions show a local alteration of the secondary structure.

**Figure 6:**
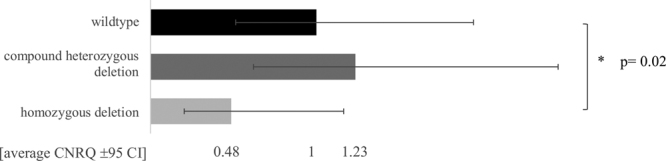
Altered expression of *TUNA* in genome-edited hESCs. Expression of *TUNA* in the wildtype, compound heterozygous and homozygous edited hESCs. Average Calibrated Normalized Relative Quantity (CNRQ) values ± 95% confidence interval (CI) is shown for 10 biological replicates of each genotype. The asterisk indicates significant difference in expression for *TUNA* (Wilcoxon rank sum test, α = 0.05).

Also on expression level an effect was observed for the naïve hESCs with the homozygous *TUNA* deletion, namely a significant decrease in expression of *TUNA* (p-value = 0.02; Wilcoxon rank sum test, α = 0.05). This is not the case for the compound heterozygous deletion where no significant difference in expression was seen compared to the naïve wildtype hESCs (p-value = 0.19, Wilcoxon rank sum test, α = 0.05) (Fig. 6).

### Efficient differentiation of naïve hESCs to neural progenitor cells

**Figure 7:**
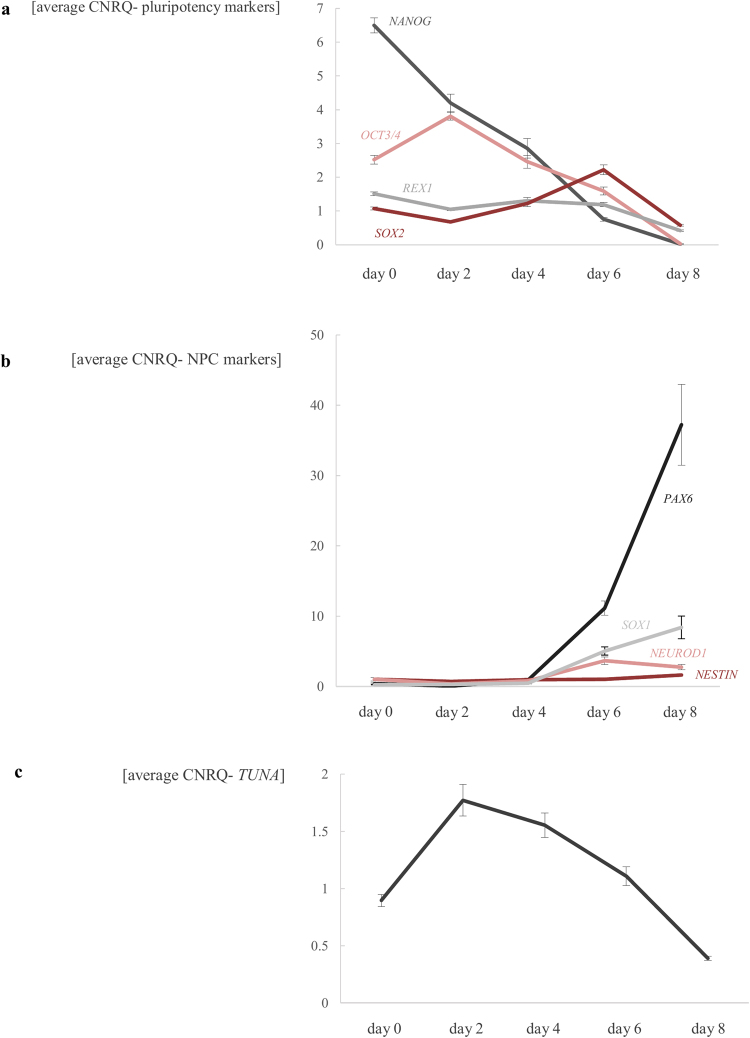
Successful differentiation of wildtype naïve hESCs to neural progenitor cells (NPCs). (**a**) Pluripotency markers (*OCT3/4*, *REX1*, *SOX2* and *NANOG*) decrease during neural differentiation of wildtype naïve hESCs, while the expression of neural progenitor cell markers (*PAX6*, *NESTIN*, *NEURDO1*, *SOX1*) increases (**b**). Expression of *TUNA* increases during first two days (**c**). RNA was sampled at day 0, 2, 4, 6 and 8 of neural induction. Average CNRQ ± standard error of three independent biological replicates is shown.

We set up a neural differentiation with STEMdiff Neural Induction Medium starting from naïve wildtype hESCs. RNA was isolated at different time points (day 0, 2, 4, 6 and 8 of neural induction) from three independent biological replicates. As shown in Fig. 7a, the expression of core-pluripotency genes *OCT3/4*, *REX1*, *SOX2* and *NANOG* decreases in time. At the same moment, the expression of the neural progenitor markers *PAX6*, *NESTIN*, *NEUROD1* and *SOX1* increases (Fig. 7b), indicating that the neural differentiation was successful.

### *TUNA*, a lncRNA involved in neural differentiation?

**Figure 8:**
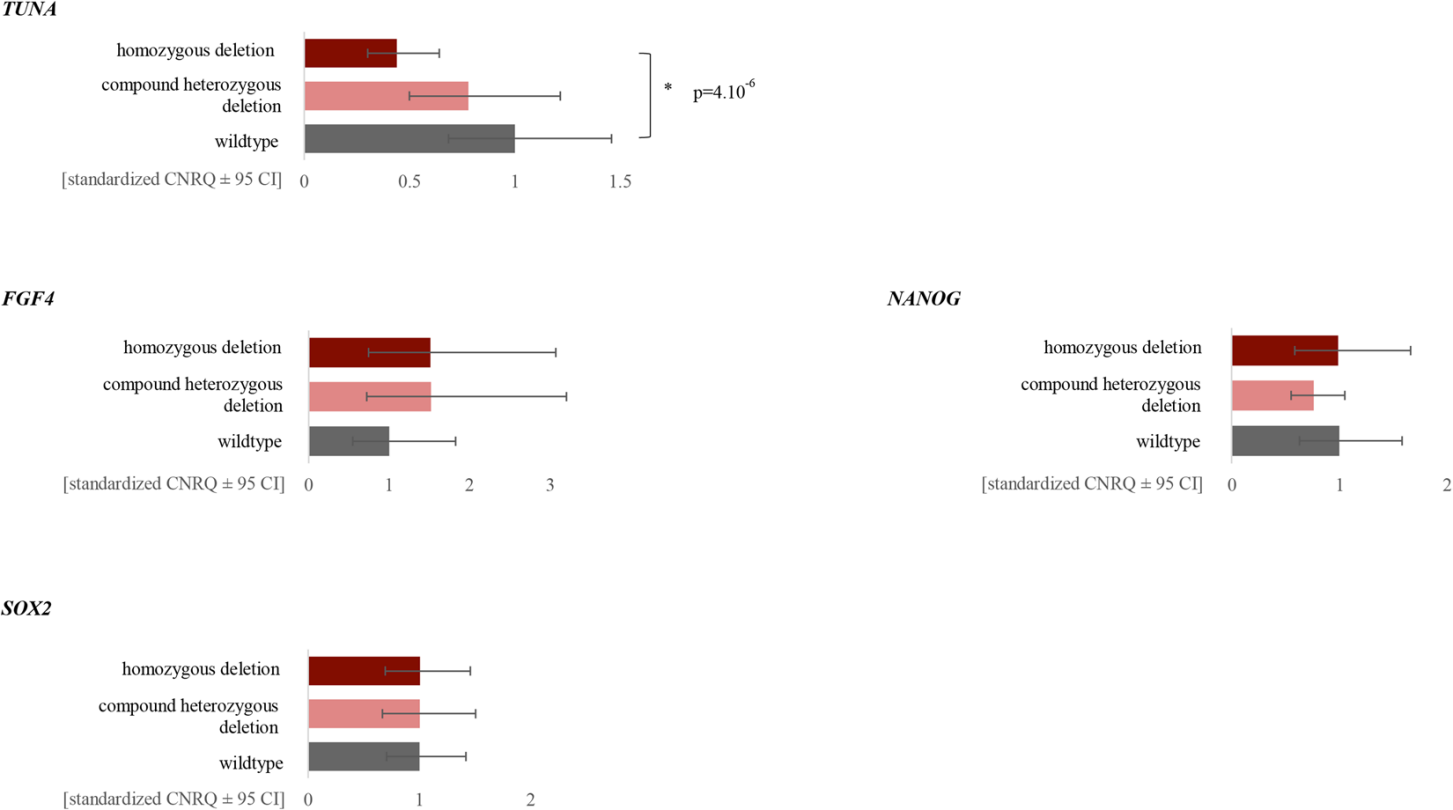
Generated indels in conserved region of *TUNA* don’t cause a significant effect on pluripotency and neural differentiation. Expression of *TUNA* and its downstream targets (*FGF4*, *NANOG*, *SOX2*) in the wildtype, compound heterozygous and homozygous edited hESCs during neural differentiation. Average standardized (according Willems *et al*.^34^) CNRQ values ± 95% confidence interval (CI) are shown for 10 biological replicates of each genotype. RNA was collected of the naïve hESCs (day 0) and at day 6 of neural induction. Significant p-values are indicated with an asterisk (Wilcoxon rank sum test with BH correction, FDR = 0.05).

We see a similar expression pattern of *TUNA* during neural differentiation as described by Lin *et al*.^28^, namely, an increase during the first two days of neural induction (Fig. 7c). To test whether knockdown of *TUNA* would interfere with the neural differentiation, as seen by Lin *et al*. after knockdown with shRNAs, the expression of the pluripotency and neural markers was monitored during differentiation of both wildtype as well as the compound heterozygous and homozygous edited stem cell lines of *TUNA* (Fig. 8, Supplemental Fig. 7). From ten independent biological replicates RNA was extracted from the naïve hESCs (day zero) and at day six after neural induction. Because ESCs are often subject to high variability in expression between biological replicates, we standardized our expression data as described by Willems *et al*.^34^. During neural differentiation, a decrease in expression of *TUNA* was observed in both genome-edited stem cell lines, which was significant for the homozygous deletion (p-value = 4.10^−6^, false discovery rate (FDR) = 0.05). In mESCs, *Nanog*, *Sox2* and *Fgf4* are described as direct target genes of *Tuna* and decreased expression of these genes was observed after knockdown of *Tuna* with shRNAs^28^. This is not the case for *SOX2*, *FGF4* and *NANOG* in both *TUNA* genome-edited hESCs. We did not observe a significant decrease in expression of the core-pluripotency genes *REX1* and *OCT3/4*. In addition, no decrease in expression of neural progenitor markers *PAX6*, *NESTIN*, *NEUROD1* and *SOX1* was noticed during differentiation in the *TUNA* genome-edited hESCs. Remarkably, a significant increased expression of *NESTIN* is observed in the compound heterozygous stem cell line (p = 1,99.10^−5^, FDR = 0.05) (Fig. 8, Supplemental Fig. 7).

## Discussion

The naïve state of hESCs can offer a better alternative for efficient genome-editing as there is no labor-intensive maintenance of the stem cell culture and exhibit increased single-cell cloning efficiency, compared to primed hESCs^7^. Based on the three tested loci, the CRISPR/Cas9 system works efficiently in naïve hESCs both with the Cas9 nuclease as well as with the Cas9 nickase (up to 19% editing efficiency). The use of a plasmid based CRISPR approach already proved its effectiveness in pluripotent stem cells and the observed editing efficiencies are similar as described in literature for iPSCs (1–25% NHEJ in iPSCs, also without any selection steps^29,35,36^). Higher efficiencies are noticed when using a selection marker (up to 79% in iPSCs^37–39^). Next to this strategy, delivery of ribonucleoproteins (RNPs) is reported as more efficient and with reduced off-target effects compared to plasmids expressing the Cas9 nuclease^40,41^. For example, Kim *et al*. reported an editing efficiency of 66% when using Cas9 RNP electroporation in H9 hESCs^42^. Therefore, it is of great interest to optimize this system in naïve hESCs and compare the editing efficiencies with the Cas9 plasmids. When only considering the off-target effects, these will also be minimized when using the double nicking system with the plasmid approach.

Although we only screened four predicted off-target sites, for one sgRNA of *EMX1* one of the screened sites showed cleavage with the wildtype Cas9 nuclease and not with the Cas9 nickase. This confirms the higher specificity of the double nicking system, as described by Ran *et al*.^24^. It also stresses the importance of screening for off-target sites when using CRISPR-Cas9, especially for certain applications (e.g. gene or cell therapy). Therefore, one can screen all predicted off-targets sites or identify the off-targets empirically using methods like GUIDE-Seq^43^, Digenome-Seq^44^, SITE-Seq^45^ and CIRCLE-Seq^46^.

In contrast to Miyaoka *et al*.^36^, for our sgRNAs we don’t see an effect of the chosen cell type on the efficiency of gene-editing (naïve hESCs and HEK-293T), however we do observe an influence of the used sgRNA and chosen target locus.

For the three observed loci, the Cas9 nickase doesn’t generate predominant indels like the Cas9 nuclease does, but the indels are larger. The latter can be beneficial to perturb the function of a lncRNA, which is not as straightforward as for protein coding genes. It’s described that small deletions within lncRNAs are unlikely to disrupt their biological activity and hence larger deletions are preferred^47^.

Lin *et al*.^28^ described a highly-conserved region of ∼200 bp in the largest exon of *TUNA* that contains a functional motif. Therefore, we chose to target this motif with CRISPR/Cas9 to disturb its function. Because of the favorable characteristics of the naïve hESCs, no problems were experienced with the monoclonal isolation and we rapidly obtained two genome-edited hESCs colonies with (a) homozygous/compound heterozygous deletion(s) in this motif.

The function of lncRNAs is probably related to their higher-order structures through interactions with partner proteins^48^. After in silico assessment with RNAplfold we see that both deletions cause a change in the local secondary structure of *TUNA*, compared to the wildtype structure. Since it’s shown by Lin *et al*.^28^ that *Tuna* modulates its function through its conserved functional domain by interacting with the RNA-binding proteins PTBP1 and hnRNP-K (but not NCL), it can be anticipated that the obtained alterations in secondary structure can have a functional effect.

Recently, Lee *et al*.^49^ showed that naïve hESCs are intrinsically predisposed to differentiate towards the neural fate and have impaired mesoderm and endoderm differentiation capacity. They compared the neural differentiation potential of naïve and primed hESCs and noticed significantly more NPCs generated from the naïve hESCs. Our group also successfully differentiated wildtype and genome-edited naïve hESCs to NPCs, which has great potential for studying neurodevelopmental disorders. After differentiating the two genome-edited *TUNA* hESCs to NPCs, both showed a decrease in expression of *TUNA* during neural differentiation (significant for the homozygous deletion). Large differences between biological replicates can be noticed and hence the method by Willems *et al*. to standardize our expression data was implemented.

A possible explanation why we don’t observe the same phenotype as Lin *et al*. might be the use of different mechanisms to generate the gene knockdown: shRNA interferes at the (post)transcript-level and CRISPR/Cas9 at the DNA-level. It might be that the generated indels with CRISPR/Cas9 are too small to generate a sufficient knockdown and subsequent a functional effect. Lin *et al*. achieves a knockdown of almost 90% in the mESCs, while only the hESCs with a homozygous deletion shows a knockdown of ~50%. It might be that only these hESC clones survived because these indels have limited effect on their pluripotency. It would be interesting to investigate whether genome-edited colonies with larger indels in the functional domain or even a deletion of the entire lncRNA will have a functional effect on pluripotency and neural differentiation and will repeat the phenotype as described in Lin *et al*.

Combining pluripotent stem cells and genome-editing, in particular the CRISPR/Cas9 technology with its broad applications, will be a powerful tool for future research. The use of naïve hESCs in comparison to primed hESCs has several advantages, therefore, we optimized a general workflow for the CRISPR/Cas9 system in naïve human embryonic stem cells. Here we showed that the system works efficiently. Next to this, we also established neural differentiation starting from naïve hESCs. In future research, this workflow can be used to study candidate genes, both coding as well as noncoding, involved in neural differentiation.

## Material and Methods

### Ethical permission

The derivation of naïve human embryonic stem cells for this study was approved by Ghent University Hospital Ethical Committee (2013/822). Genome-editing of naïve human embryonic stem cells with CRISPR/Cas9 was approved by Ghent University Hospital Ethical Committee (2017/0923). All experiments were performed in accordance with relevant guidelines and regulations.

### Generation and culture of hESC

In-house derived primed and naïve hESCs were cultured as described^6^. The naïve hESCs were maintained in 20% O_2_ and 5% CO_2_ conditions instead of hypoxic conditions (5% O_2_) and 6% CO_2_. In brief, the naïve hESCs were cultured on inactivated Mouse Embryonic Fibroblasts (MEFs) (GSC-6201G, Tebu-Bio) in naïve hESC medium consisting of KO-DMEM (10829018, Invitrogen) containing 20% knockout serum replacement (10828028, Invitrogen), 1% non-essential amino acids (11140050, Invitrogen), 1% Penicillin/Streptomycin (15140122, Invitrogen), 0.1 mM L-glutamine (25030024, Invitrogen) and 0.1 mM β-mercaptoethanol (31350010, Invitrogen). This naïve hESC medium is supplemented with 12 ng/ml bFGF (100–18B, Peprotech), 1000U recombinant human LIF (L5283, Sigma) and the small molecules 1 µM PD0325903 (13034, Sanbio), 3 µM CHIR99021 (1386, Axon Medchem), 10 µM Forskolin (F6886, Sigma) and 50 ng/ml ascorbic acid (A8960, Sigma). The naïve colonies were passaged as single cells using 0.05% trypsin/EDTA (25300054, Invitrogen) every three days and were re-plated on inactivated MEFs.

### Design and construction CRISPR plasmids

CRISPR plasmids were constructed for three target genes: *TUNA*, *MEG3* and *EMX1*. sgRNAs were designed in the largest exon of *TUNA*, which is highly conserved^28^, and in the promotor region of *MEG3*. For *EMX1*, sgRNAs 9 and 10 from Ran *et al*.^24^ were used (Supplemental Table 3). For each gene, two guide RNAs were designed to allow double nicking using the CRISPR/Cas9 design tool developed by the Zhang lab at MIT (crispr.mit.edu). For the selected sgRNA target sequence, the recognition site for BbsI was added to the sequence and the single stranded DNA molecules were ordered at Integrated DNA Technologies (IDT, Belgium): 5′-CACC-(G)-N_20_-3′ (forward) and 3′-(C)-N_20_-CAAA-5′ (reverse). If the target sequence does not start with a G, add a G and a C to respectively the forward and reverse oligonucleotide. Both oligonucleotides were resuspended to a concentration of 100 µM, mixed in equimolar concentration, heated for 2 minutes at 95 °C, cooled down to room temperature and diluted to 0.04 µM.

#### Cas9 nuclease

The two sgRNAs used with the Cas9 nickase were individually cloned into the pX330 plasmid (Addgene Plasmid #42230). The vector was digested with BbsI (R0539S, Bioké) and annealed oligonucleotides were ligated into the digested plasmid with the DNA ligation kit (TB6023, Westburg) according manufacturer’s instructions.

#### Double nicking D10A Cas mutant

Both sgRNAs were first individually cloned into the pX335 plasmid (Addgene Plasmid #42335). To enhance transfection efficiency, both guide RNAs were then cloned in the same pX335 plasmid. Therefore, the U6-sgRNA2 cassette was amplified using primers containing the XbaI and KpnI sites and cloned into the pX335 plasmid containing the U6-sgRNA1 cassette.

### Determine off-target sites

The CRISPR/Cas9 design tool from the Zhang lab gives a ranked list of possible off-target sites for each sgRNA, based on the number and position of mismatches. For each sgRNA the top four ranked off-targets were screened for presence of undesired indels by next-generation-sequencing. Supplemental Table 4 contains the primer sequences and the genomic positions (hg19).

### Transfection of CRISPR plasmids into naïve human embryonic stem cells

Naïve hESCs cultured on MEFs were dissociated into single cells using 0.05% Trypsin/EDTA. 2.10^5^ cells were nucleofected with 0.4 µg plasmid in 20 µl P3 Primary Cell Nucleofector Solution with program DN100 (4D Nucleofector, Lonza), according to the manufacturer’s instructions. For every transfection, a mock-treated control was included (cells were exposed to the Nucleofector solution and subjected to the Nucleofection procedure, but in the absence of DNA). The transfected cells were subsequently plated onto fresh inactived MEFs and cultured in naïve human embryonic stem cells medium, supplemented with 10 µM Rock inhibitor (S1049, Selleckchem). To test overall editing efficiency of the CRISPR, DNA was isolated from a pool of cells two days after nucleofection.

### Transfection of CRISPR plasmids into HEK-293T cells

2.10^5^ cells HEK-293T cells were nucleofected with 0.4 µg plasmid in 20 µl SF Primary Cell Nucleofector Solution with program CM-130 (4D Nucleofector, Lonza), according to the manufacturer’s instruction. For every transfection, a mock-treated control was included (cells were exposed to the Nucleofector solution and subjected to the Nucleofection procedure, but in the absence of DNA). To test overall editing efficiency of the CRISPR, DNA was isolated from a pool of cells two days after nucleofection.

### Isolation of clonal cell lines by limiting dilution

Two days after nucleofection, the transfected naïve hESCs were dissociated into single cells using 0.05% Trypsin/EDTA. To prevent clumping of cells, a cell strainer was used. The cells were diluted to a final concentration of 0.5 cells per 100 µl of naïve medium, supplemented with 10 µM Rock inhibitor (S1049, Selleckchem), to reduce the likelihood of having multiple cells per well. 100 µl of diluted cells were added to each well of a 96-well plate, coated with inactivated MEFs. After six to eight days, individual colonies were observed and these were picked and further expanded.

### Genomic DNA extraction

Genomic DNA was extracted with the QuickExtract DNA isolation kit (QE09050, Epicentre) according to the manufacturer’s instructions. Depending on the number of cells, 50–100 µl of the solution was used. Genomic DNA was diluted to 20–25 ng/µl and 2 µl was used as input for PCR amplification of the desired target site.

### Next-generation-sequencing (NGS) and data analysis

Extracted DNA was singleplex PCR-amplified with the Kapa2G Robust HotStart Ready Mix (KK5702, Kapa Biosystems). PCR products were subsequently sequenced on an Illumina MiSeq platform according to De Leeneer *et al*.^50^. For amplicons from DNA extracted from pools of cells, BATCH-GE generated a detailed overview on genome editing efficiency and the detected indel variants after NGS^51^. The region of interest for Cas9 nuclease and nickase was respectively defined as [theoretical cleavage site ± 30 bp] and [theoretical cleavage site sgRNA1–30 bp; theoretical cleavage site sgRNA2 + 30 bp]. See Supplemental Table 4 for primers.

### RNA isolation, cDNA synthesis and quantitative PCR

Total RNA was extracted from cells using TRIzol reagent (15596026, Invitrogen) and the miRNeasy Mini RNA extraction kit (217004, Qiagen) according to the manufacturer’s instructions. Quality assessment of RNA was performed with the High Sensitivity RNA Analysis Kit on a Fragment Analyser (DNF-472-0500, Advanced Analytical Technologies). Complementary DNA synthesis was performed with the iScript cDNA synthesis kit (170-8891, Bio-Rad Laboratories) in accordance with the manufacturer’s instructions. Quantitative reverse transcription-polymerase chain reaction was performed using 2x SYBR Green SsoAdvanced Supermix (172-5274, BioRad Laboratories), 5 µM forward and reverse primers, and 5 ng complementary DNA as input. For all experiments, expression was normalised using three reference genes (*ACTB*, *TBP* and *YWHAZ*) and evaluated with qBasePlus software (Biogazelle). See Supplemental Table 5 for primers used for qPCR.

### Analysis expression data and statistical testing

Statistical differences between groups were evaluated by Wilcoxon rank sum test (alpha level = 0.05). For the neural differentiation experiment, in every group (wildtype, homozygous deletion, heterozygous deletion) 10 independent biological replicates were included. We standardized our expression data from the neural differentiation experiment as described by Willems *et al*.^34^ (performing series of sequential corrections including log transformation, mean centering and autoscaling) to reduce the variability between biological replicates. Benjamini-Hochberg (BH) correction was used to account for multiple comparison testing (false discovery rate = 0.05).

### Neural differentiation of naïve human embryonic stem cells

Naïve human embryonic stem cells were differentiated to neural progenitor stem cells with STEMdiff Neural Induction Medium (05835, STEMCell Technologies) according to manufacturer’s instructions with some adaptations. Naïve hESCs, cultured on MEFs, were dissociated to single cells with trypsin/EDTA. To reduce the concentration of MEFs in the cell suspension, the cell suspension was seeded on 0.1% gelatin coated flasks (G-1890, Sigma-Aldrich) and incubated for 1 hour at 37 °C. Since the MEFs will attach to the gelatin first, the remaining suspension of unattached cells was collected. 7.10^4^–8.10^4^ cells were resuspended in STEMdiff Neural Induction Medium + 10 µM Rock-Inhibitor (S1049, Selleckchem) and plated on Poly-L-Ornithine/Laminin coated 12-well plates. Medium was daily changed until the desired time point. 10 µM Rock-Inhibitor was added at day 0 and day 1.

### Data availability

All data generated or analyzed during this study are included in this published article (and its Supplementary Information files). The raw datasets generated and/or analyzed are available from the corresponding author on reasonable request.

## Electronic supplementary material

Supplemental information
