# Supplementary material for: CRISPR/Cas9-mediated genome editing in naïve human embryonic stem cells
Source: Sci Rep. 2017 Nov 30;7:16650. doi: 10.1038/s41598-017-16932-y (PMC5709416; doi:10.1038/s41598-017-16932-y)
Supplement: Supplementary file 1 — Supplemental information [file 41598_2017_16932_MOESM1_ESM.pdf]

Supplemental Information

CRISPR/Cas9-mediated genome editing in naïve human embryonic stem cells

Eva Z. Jacobs[1], Sharat Warrier[2], Pieter-Jan Volders[1], Eva D'haene[1], Eva Van Lombergen[1], Lies Vantomme[1], Margot Van der Jeught[2], Björn Heindryckx[2], Björn Menten[1], Sarah Vergult[1*]

Figure legends Supplemental Figures and Tables

Supplemental Figure 1: **Similar editing efficiencies for HEK-293T cells and naïve hESCs**

Editing efficiencies for *TUNA*, *EMX1* and *MEG3* after transfecting pX335, pX330-sgRNA1 and pX330-sgRNA2 plasmids in HEK-293T cells. As a control, mock-treated HEK-293T cells (no plasmid) are used. Grey dots represent two biological replicates, the asterisk indicates the average of both replicates.

Supplemental Figure 2: **Same predominant indels are present in naïve hESCs and HEK-293T cells**

Distance of the start position of each indel to the theoretical cleavage site (= 3 bp upstream of the PAM sequence) is presented on the x-axis, while the relative frequency of the indel is presented on the y-axis for **a**) nuclease – sgRNA1 and **b**) nuclease – sgRNA2. Length of indels is represented by the different colors. Predominant indels are indicated with an arrow. Only one replicate is shown.

Supplemental Figure 3: **Majority of indels is present between both cleavage sites after editing with Cas9 nickase**

Distance of the start position of each indel to the theoretical cleavage site (= 3 bp upstream of the PAM sequence) is presented on the x-axis, while the relative frequency of the indel is presented on the y-axis after editing with the Cas9 nickase. Length of indels is represented by the different colors. Only one replicate is shown.

Similar as for the naïve hESCs, in the HEK-293T cells the majority of indels is present between the two theoretical cleavage sites (indicated by the black lines at position 0 (sgRNA1) and at positions 44 (*TUNA*), 25 (*EMX1*) and 38 bp (*MEG3*). Percentages of indels 1) within or spanning both the two cleavage sites and 2) indels spanning at least one of the two sites are shown below the graph.

Supplemental Figure 4: **Off-targets detected with Cas9 nuclease, not with Cas9 nickase**

After analysis with BATCH-GE and visualization of the sequencing reads in IGV (chr10:94603018-94603100 (hg19)), indels were detected at an intron of *EXOC6* (one of the off-target sites of sgRNA2 of *EMX1*) after transfection with the Cas9 nuclease (editing efficiency of 12%). No indels were observed after transfection of the Cas9 nickase.

Supplemental Figure 5: **Successful generation of monoclonal genome-edited *TUNA* colonies**

Sequencing reads are visualized in IGV (region: chr14:96,389,236-96,389,314 (hg19)) after monoclonal isolation. The region of interest in a wildtype sample is shown in **a**), while **b**) and **c**) respectively show the compound heterozygous deletions and the homozygous deletion. Positions of sgRNA1 and sgRNA2 are indicated with respectively a dark grey and light grey line.

Supplemental Figure 6: **Naïve human embryonic stem cells maintain the naïve state after genome-editing**

a) *DNMT3B* shows increased expression in the primed hESCs, compared to both naïve genome-edited hESCs and wildtype naïve hESCs. For *KLF2* and *TCL1B*, a trend of increased expression in the naïve hESCs is observed. *NANOG* shows similar expression in naïve and primed hESCs. Expression of each biological replicate (CNRQ) is represented by a dot (n=3 (primed), n=4 (naïve cells with homozygous deletion), n=5 (naïve wildtype and naïve cells with compound heterozygous deletion). Average CNRQ expression is shown as a horizontal line for each group.

b) No differences in morphology were observed between both genome-edited (top) and wildtype naive hESCs (bottom, left). Next to this, the domed-shaped naïve colonies can be distinguished from the flattened primed colonies (bottom, right). Scale bar represents 1000 um.

Supplemental Figure 7: **Generated indels in conserved region of *TUNA* don't cause a significant effect on pluripotency and neural differentiation**

Expression of pluripotency markers (*OCT3/4* and *REX1*) and neural progenitor markers (*NESTIN*, *PAX6*, *SOX1* and *NEUROD1*) in the wildtype and both *TUNA* genome-edited hESCs during neural differentiation. Average standardized (according Willems et al. [34]) CNRQ values ± 95% confidence interval (CI) are shown for 10 biological replicates of each genotype. RNA was collected from the naïve hESCs (day 0) and at day 6 of neural induction. Significant p-values are indicated with an asterisk (Wilcoxon rank sum test with BH correction, FDR= 0.05).

Supplemental Table 1: **Cas9 nickase generates larger indels compared to wildtype Cas9 nuclease in naïve hESCs**

Indel characteristics of Cas9 nickase (pX335 plasmid) and Cas9 nuclease (pX330 plasmid) in naïve hESCs: amount (n) and median size of deletions and insertions.

Supplemental Table 2: **Cas9 nickase generates larger indels compared to wildtype Cas9 nuclease in HEK-293T cells**

Indel characteristics of Cas9 nickase (pX335 plasmid) and Cas9 nuclease (pX330 plasmid) in HEKT-293 cells: amount (n) and median size of deletions and insertions.

Supplemental Table 3: **sgRNA sequences for CRISPR plasmid design**

sgRNAs for *TUNA* and *MEG3* are designed with crispr.mit.edu and for *EMX1*, sgRNA9 and sgRNA10 were used from Ran *et al* [24].

Supplemental Table 4: **Primers used for amplification on- and off-targets (hg19**)

Supplemental Table 5: **Primers used for qPCR**

qPCR primers for *SOX2* and *FGF4* were purchased from Bio-Rad (respectively assay ID qHsaCED0036871 and qHsaCID0020331).

Supplemental Figure 1

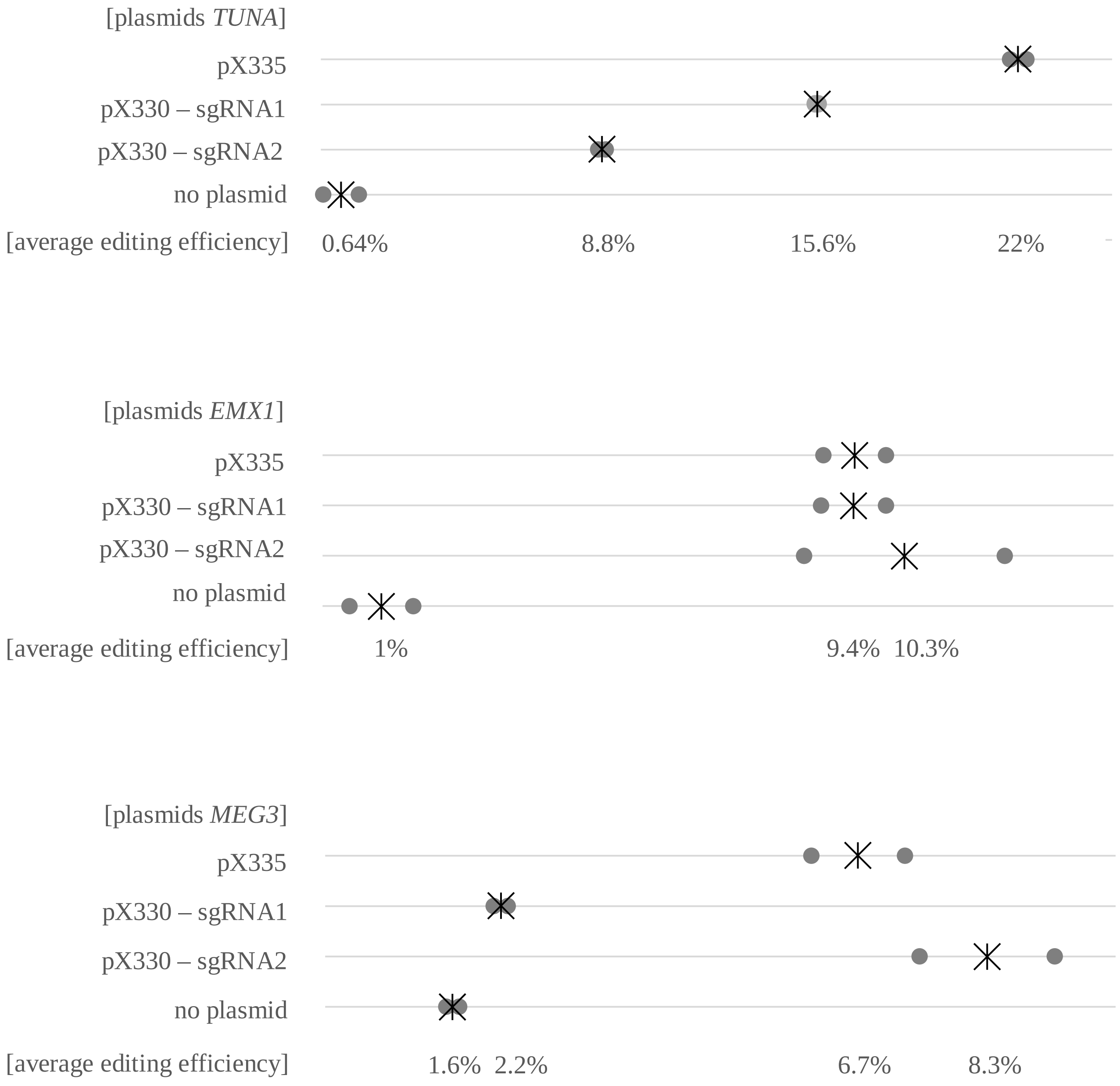

Supplemental Figure 2

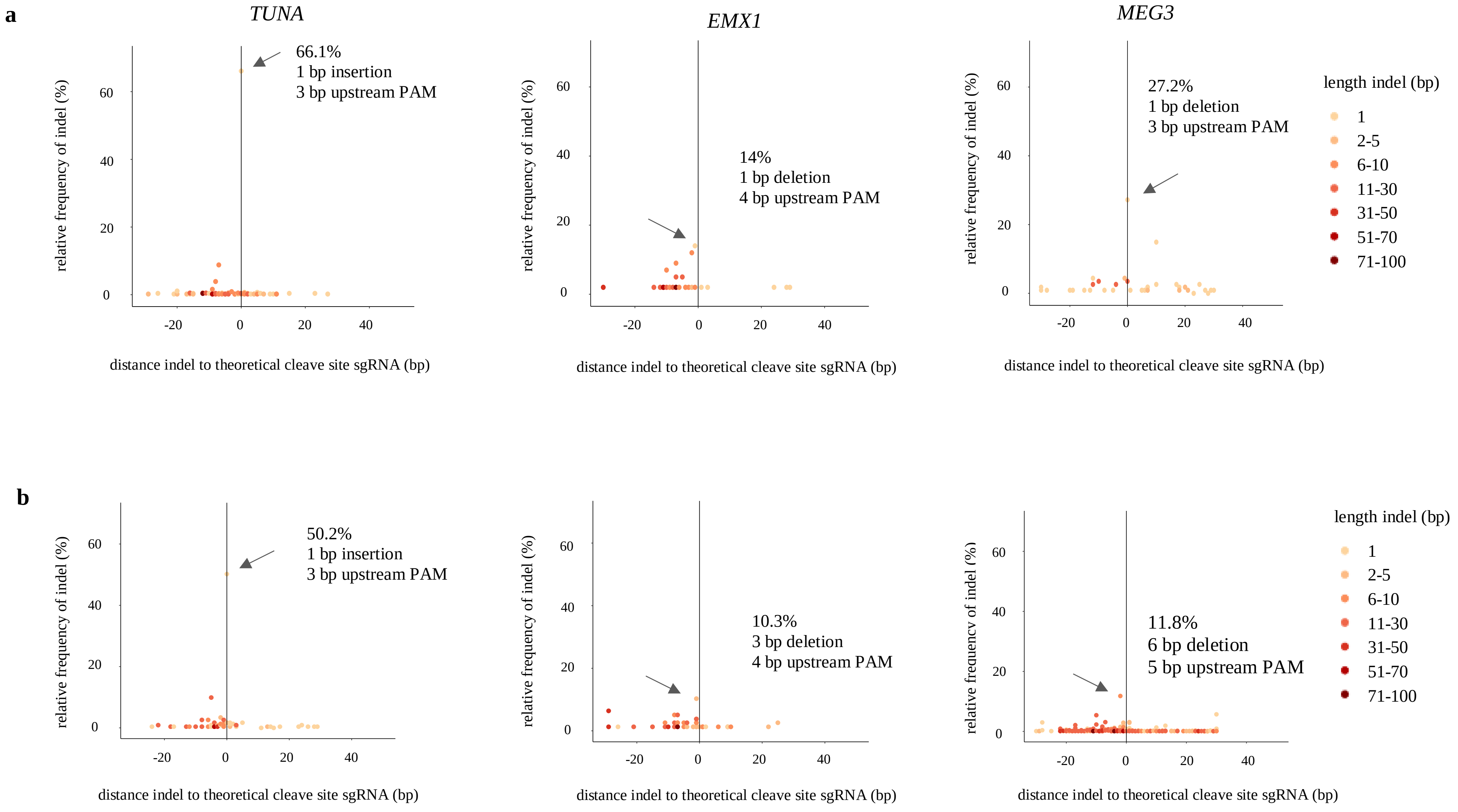

Supplemental Figure 3

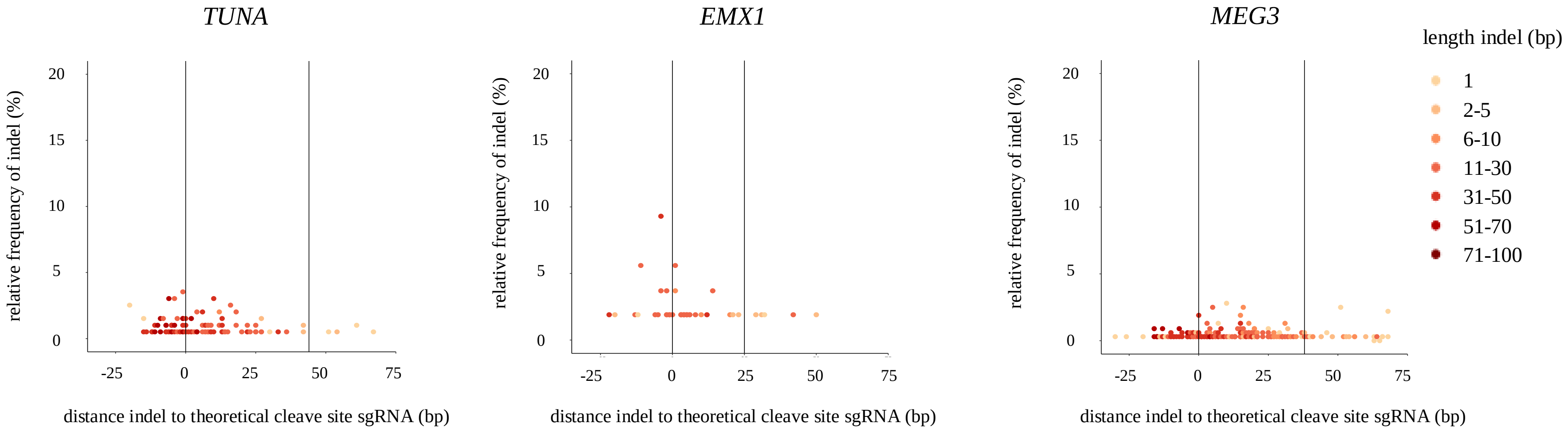

	% indels between both cleavage site	% indels overlapping one of both cleavage sites
replicate 1	52	93
replicate 2	48	94

	% indels between both cleavage site	% indels overlapping one of both cleavage sites
replicate 1	57	85
replicate 2	62	90

	% indels between both cleavage site	% indels overlapping one of both cleavage sites
replicate 1	56	86
replicate 2	57	75

Supplemental Figure 4

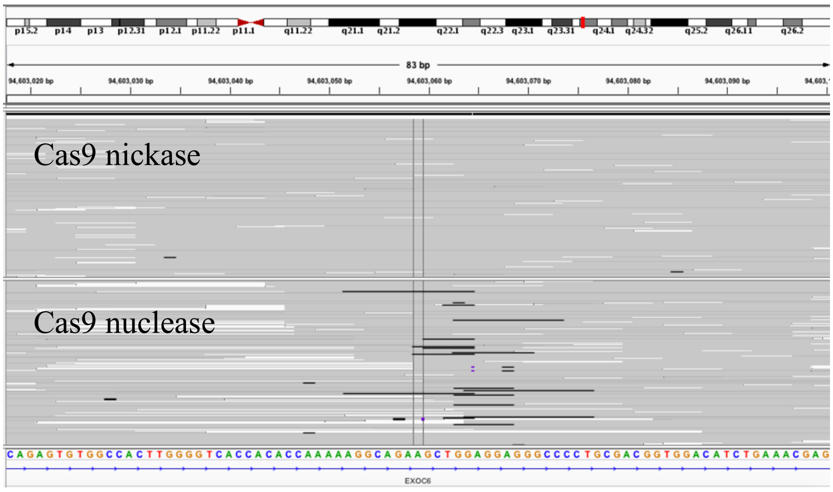

Supplemental Figure 5

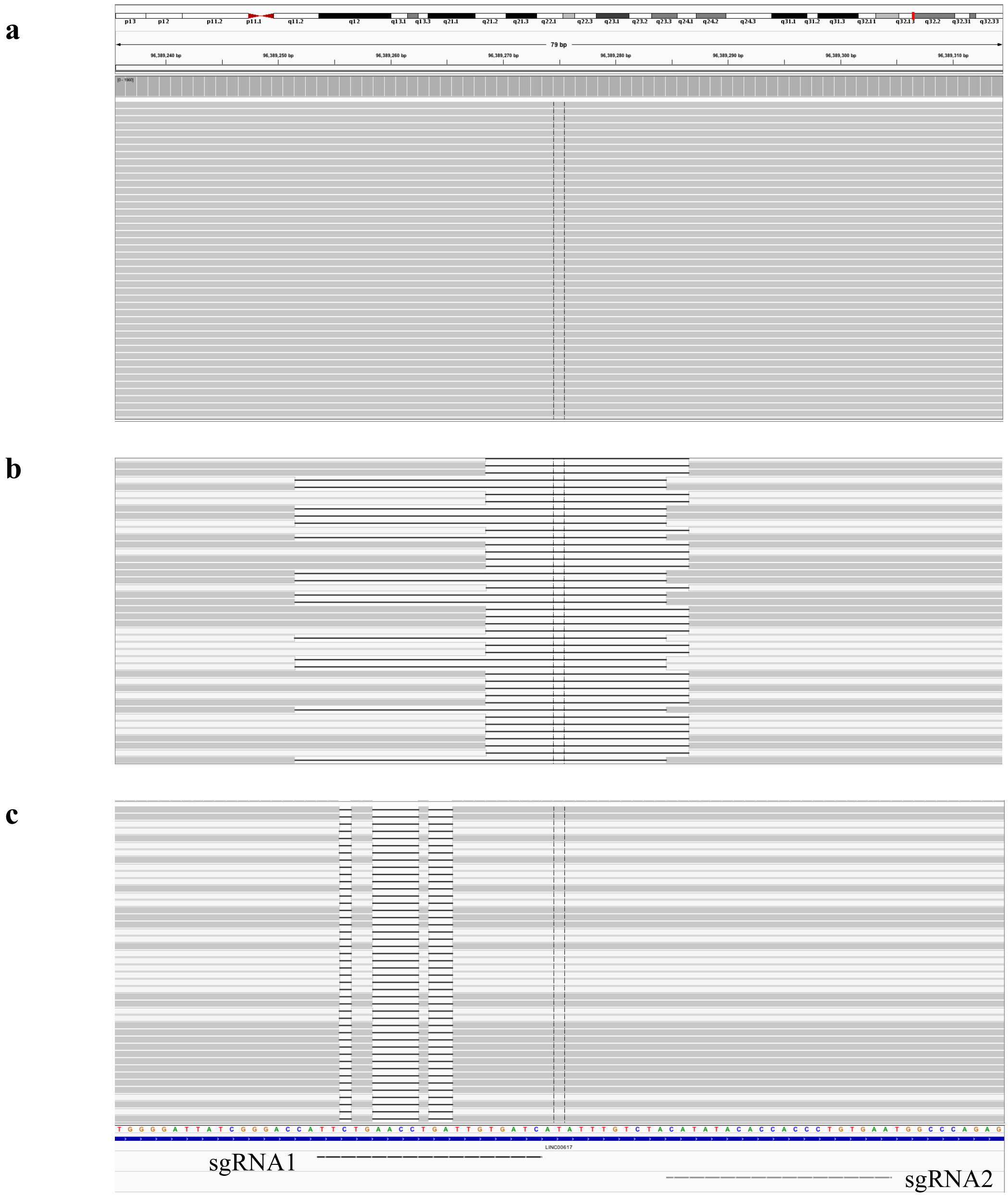

Supplemental Figure 6

a

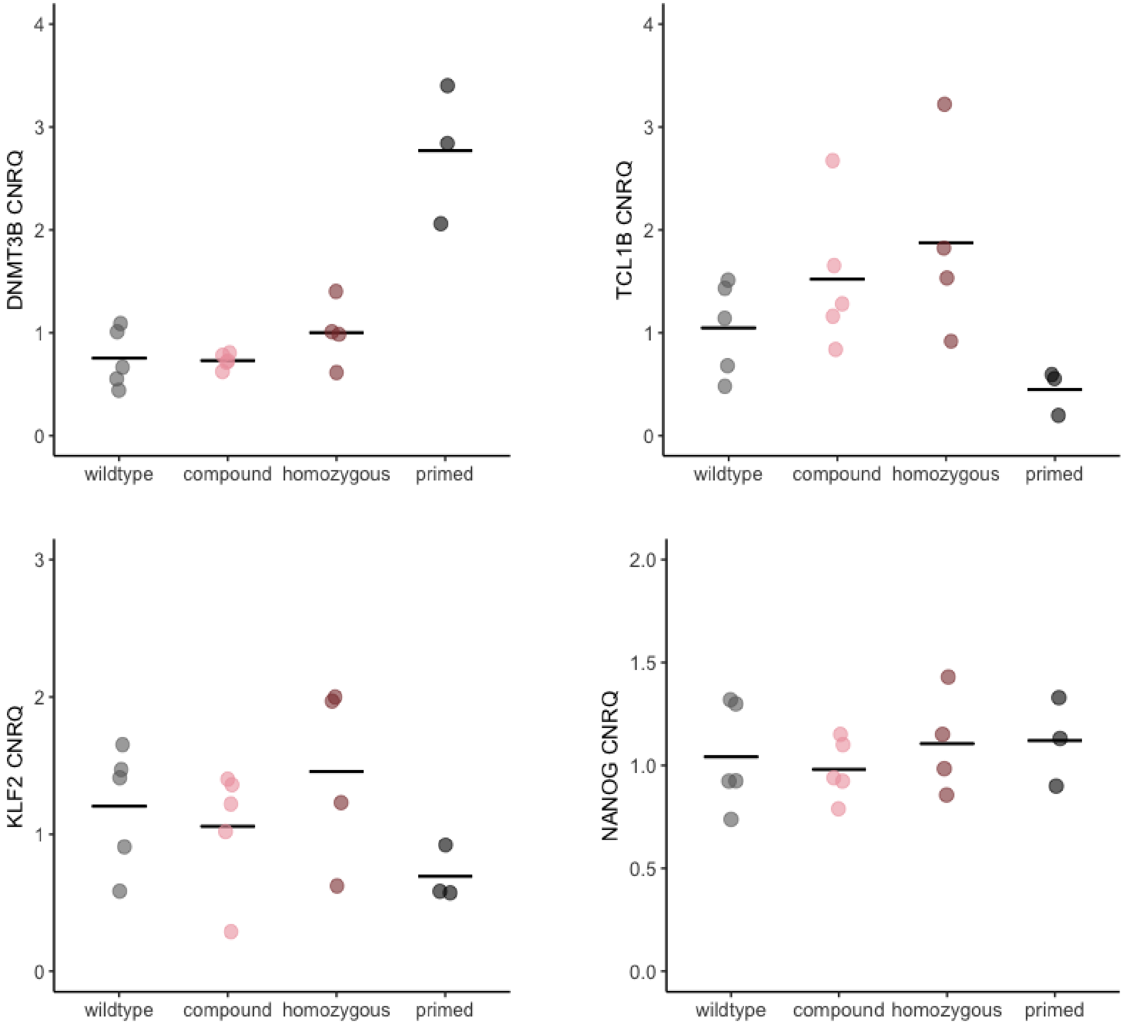

b

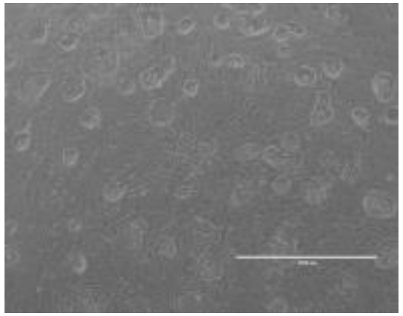

genome-edited naïve hESCs

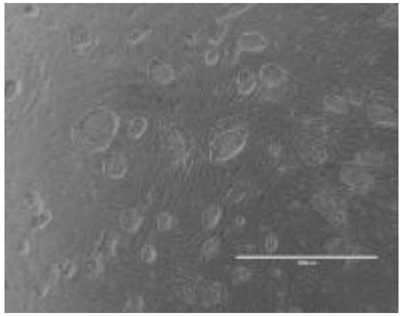

genome-edited naïve hESCs

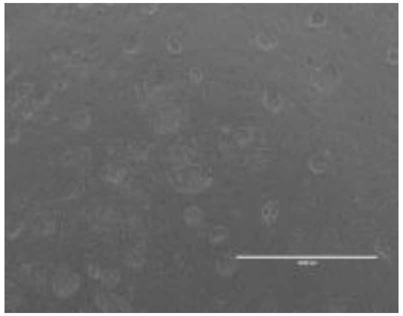

wildtype naïve hESCs

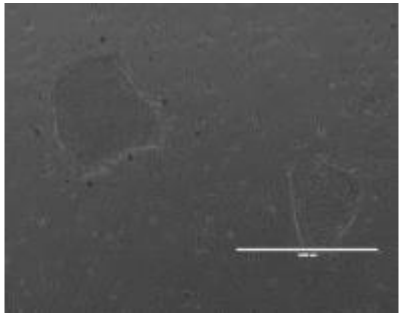

primed hESCs

Supplemental Figure 7

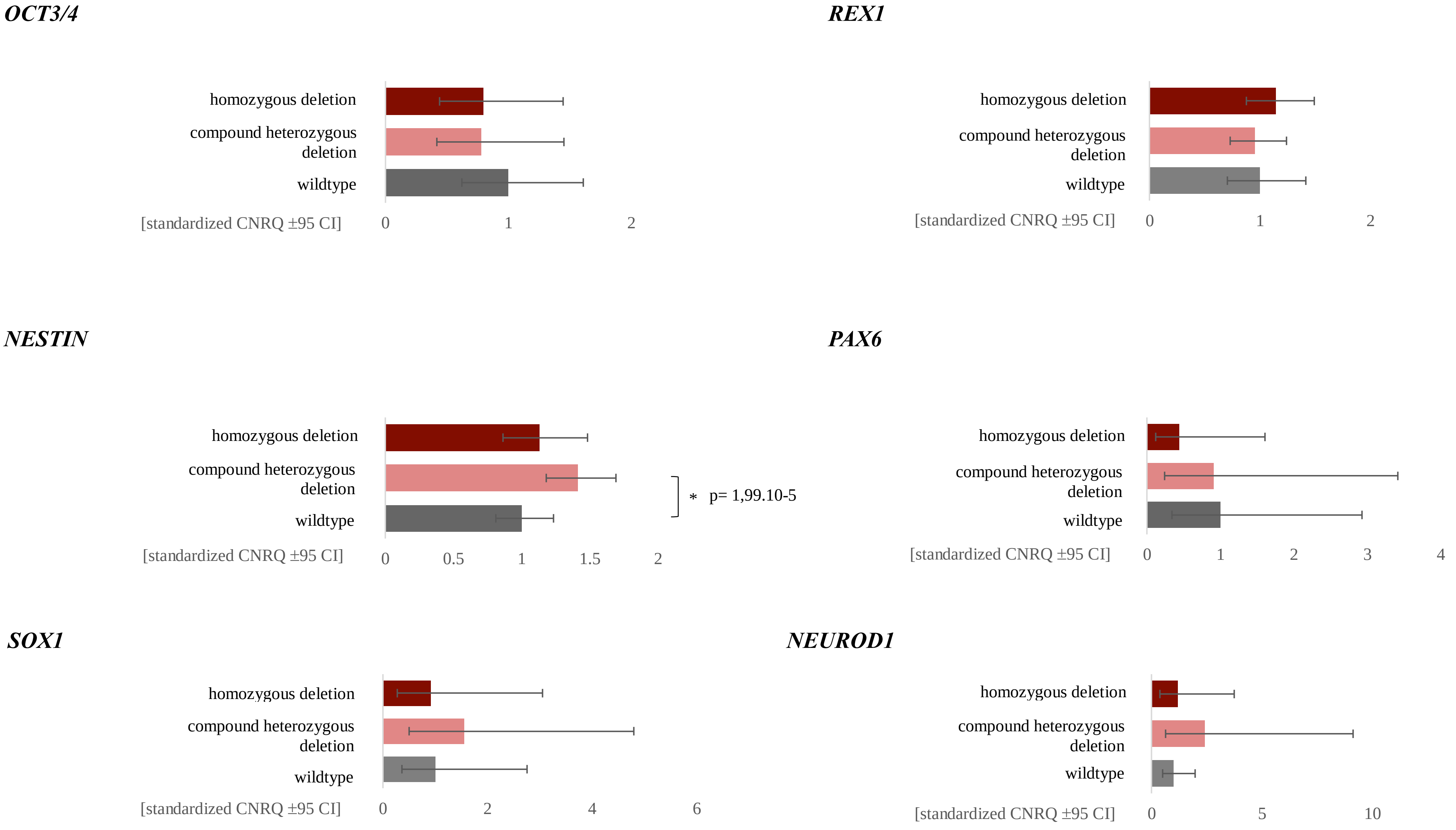

Supplemental Table 1

TUNA

plasmid	replicate	deletions			insertions		
		n	%	median	n	%	median
pX335	1	432	72	21	166	28	13
	2	450	73	17	164	27	12
pX330 sgRNA1	1	154	31	9	346	69	1
	2	272	33	9,5	546	67	1
pX330 sgRNA2	1	66	27	6	178	73	1
	2	90	38	2	146	62	1
none	1	10	83	1	2	17	1
	2	20	95	1	1	5	1

EMX1

plasmid	replicate	deletions			insertions		
		n	%	median	n	%	median
pX335	1	70	73	7	26	27	4
	2	76	88	3	10	12	14
pX330 sgRNA1	1	148	81	6	34	19	7
	2	86	83	3	18	17	6
pX330 sgRNA2	1	108	86	8	18	14	1
	2	146	87	3	22	13	3
none	1	10	91	1	1	9	1
	2	16	89	1	2	11	2,5

MEG3

plasmid	replicate	deletions			insertions		
		n	%	median	n	%	median
pX335	1	34	77	15	10	23	25
	2	56	47	1	62	53	5
pX330 sgRNA1	1	10	71	1	4	29	1
	2	14	54	1	12	46	1
pX330 sgRNA2	1	48	100	8	0	0	/
	2	188	82	12	42	18	1
none	1	13	93	1	1	7	1
	2	17	100	1	0	/	/

Supplemental Table 2

TUNA

plasmid	replicate	deletions			insertions		
		n	%	median	n	%	median
pX335	1	368	93	29	28	7	13
	2	580	92	32	50	8	7
pX330 sgRNA1	1	330	29	10	802	71	1
	2	800	29	10	1922	71	1
pX330 sgRNA2	1	194	42	12	272	58	1
	2	448	37	12	750	63	1
none	1	48	96	1	2	4	1
	2	54	82	1	12	18	1

EMX1

plasmid	replicate	deletions			insertions		
		n	%	median	n	%	median
pX335	1	94	87	16	14	13	4
	2	84	84	12	16	16	4
pX330 sgRNA1	1	84	98	8	2	2	3
	2	64	91	3.5	6	9	1
pX330 sgRNA2	1	114	92	4	10	8	1
	2	136	87	9	20	13	3
none	1	8	100	2	0	0	/
	2	4	100	1	0	0	/

MEG3

plasmid	replicate	deletions			insertions		
		n	%	median	n	%	median
pX335	1	526	83	17	110	17	10
	2	628	84	10	124	16	10,5
pX330 sgRNA1	1	146	64	1	82	36	1
	2	38	79	1	10	21	1
pX330 sgRNA2	1	1794	90	6	194	10	1
	2	532	83	5	108	17	1
none	1	34	71	1	14	29	1
	2	24	100	1	0	0	/

Supplemental Table 3

Gene ID	**sgRNA sequence 1** + PAM<br>**sgRNA sequence 2** + PAM	Strand	Target sequence	Distance
EMX1	**GCCGTTTGTACTTTGTCCTC**CGG<br>**CAAACGGCAGAAGCTGGAGG**AGG	Antisense<br>Sense	**GAGGACAAAGTA**CAAACGGC**AGAAGCTGGAGG**	8 bp overlap
TUNA	**GATCACAATCAGGTTCAGAA**TGG<br>**CATATACACCACCCTGTGAA**TGG	Antisense<br>Sense	**TTCTGAACCTGATTGTGATC**ATATTTGTCTA**CATATACACCACCCTGTGAA**	11 bp spacer
MEG3	**GCTTTTTCCCTGCGTGAGCC**CGG<br>**GCGACCACAGGGTGTTGGTCA**TGG	Antisense<br>Sense	**GGCTCACGCAGGGAAAAAGC**ACCC**GCGACCACAGGGTGTTGGTCA**	4 bp spacer

Supplemental Table 4

On-targets	
TUNA	F: AGGCTCCTCTCTCTGTACCA
	R: TGAATTTCTGCACCCATCCG
EMX1	F: CCATCCCCTTCTGTGAATGT
	R: GGAGATTGGAGACACGGAGA
MEG3	F: GAGCTCTGTCTCCCATGTCA
	R: CAGCAGGACCCAGGATCAG

Off-targets			
TUNA	sgRNA1-OFF1	chr2:83402789-83402811	F: TAGCACTGCCATCATCACCT
			R: CAAGAAGCCTTGCACTTTCA
	sgRNA1-OFF2	chr20:22528567-22528589	F: GAAAACCATTGCTCTCTGGAA
			R: TCTGGAATTTTCCCTGGAATC
	sgRNA1-OFF3	chr6:21103031-21103053	F: TGTTTTCATCTTGCACCTCTTG
			R: TCGTGGTAAGAAGCCAAAAGA
	sgRNA1-OFF4	chr6:127212429-127212451	F:TGAAGATAAACAGAGATTGCTGATG
			R: CCCATGTTGCAGGAGTTACA
	sgRNA2-OFF1	chr6:127212429-127212451	F: GGGAACAACTGGTCCAGAAA
			R: TGACAGACATGCTTCCCTTAGA
	sgRNA2-OFF2	chr5:6902400-6902422	F: CCACTTGGAAAGGCTGAAAA
			R: TGCTTCTAGTTGTGGTGTCTCTG
	sgRNA2-OFF3	chr10:67239041-67239063	F: TTTTGGAAGCAGTGTGAGAGA
			R: CTGGAGCAAATTGTGGTCTG
	sgRNA2-OFF4	chr17:42467779-42467801	F: GCTGTTCAGTTGAAGGTAGGC
			R: TTCCGCTTACCGAGAGAAAA
EMX1	sgRNA1-OFF1	chr9:104480740-104480762	F: GGAGCACAGGGTTTGGTCTA
			R: AAGTTTCTCACTATGTGATTCAGTGTT
	sgRNA1-OFF2	chr11:40321937-40321959	F: GAAAGGGGAGGTGCTACTGA
			R: GCATCCTCAGATTTTGCAGTC
	sgRNA1-OFF3	chr12:2024617-2024639	F: CCCTCCTCCTTGTCCCTAAA
			R: GCCTACACAGAGGGTGAGGT
	sgRNA1-OFF4	chr3:2639019-2639041	F: AAGACCAGGTCACCCAAACTT
			R: GGGAACAGCTGGGTTACAGA
	sgRNA2-OFF1	chr10:119307611-119307633	F: TTCTGTTCCACCACGCACTA
			R: CGATTCCCTCTCCCTTTCTC

	sgRNA2-OFF2	chr10:94603048-94603070	F: ACTGCGTAGGTCACACACCA
			R: TCTCGATCTCTTGACCTTGTGA
	sgRNA2-OFF3	chr10:13752484-13752506	F: ATGAAGAACCCCAGAGCAGA
			R: TTTCGCAGAGACGGAGTTTT
	sgRNA2-OFF4	chr6:69783156-69783178	F: TGGATCTATGCCGTCTGTGA
			R: AGCCAGCTAGCAGGACTCTG
MEG3	sgRNA1-OFF1	chr12:131524286-131524308	F: TGCAGGGAGCTCTGACAAG
			R: GCCCTTGGTGTCTGTTTTGT
	sgRNA1-OFF2	chr4:3250616-3250638	F: CAGCCCCAGTTCAGAAGG
			R: TGATGTCACGTTTCCATAGCA
	sgRNA1-OFF3	chr17:46201158-46201180	F: ACAGCAGGAGCCATTCAAAG
			R: TCAGTCATCCTGGGTAATGGA
	sgRNA1-OFF4	chr17:67323433-67323455	F: GTTGACTCAGTGCGGGTGA
			R: AGAAAGAGCGTGGCGAGAT
	sgRNA2-OFF1	chr2:124430470-124430492	F: GGCCATGGCTGAAATAAATC
			R: TTTTGGCAAACTCTTTAAAGCTAC
	sgRNA2-OFF2	chr6:136127143-136127165	F: TGAAGAAGCAGGAAGAAGAAATG
			R: AGTGAGCTGTGATTGCACCA
	sgRNA2-OFF3	chr5:136390866-136390888	F: GACCACAGAGACCACAATGG
			R: GCTTAAAACAATACCCCCAGA
	sgRNA2-OFF4	chr18:76477199-76477221	F: GCACCTATCTAAAAGGCAGACA
			R: AACTGCGGGGTCAGTCAT

Supplemental Table 5

TUNA	F: CCTCCGGATGCGCTTCTC
	R: CGATAATCCCCAGCATTGCC
OCT3/4	F: GACAGGGGGAGGGGAGGAGCTAGG
	R: CTTCCCTCCAACCAGTTGCCCCAAAC
REX1	F: CAGATCCTAAACAGCTCGCAGAAT
	R: ATGAATGAGGAGATGCTTTCTCAGG
NANOG	F: GATTTGTGGGCCTGAAGAAAACT
	R: AGGAGAGACAGTCTCCGTGTGAG
PAX6	F: CATTTGGCCCTTCGATTAGA
	R: AATTGAGGCCCTGGAGAAA
NESTIN	F: CGCACCTCAAGATGTCCCTC
	R: CAGCTTGGGGTCCTGAAAGC
NEUROD1	F: AAGACGCAGAAGCTGTCCAA
	R: AGCGTCTGAACGAAGGAGAC
SOX1	F: AACACTTGAAGCCCAGATGGA
	R: GCAGGCTGAATTCGGTTCTC
